# Data on genotype frequency for SNPs associated to age of smoking onset and successful smoking cessation treatment

**DOI:** 10.1016/j.dib.2019.103893

**Published:** 2019-04-17

**Authors:** Gloria Pérez-Rubio, Luis Alberto López-Flores, Alejandra Ramírez-Venegas, Raúl H. Sansores, Ramcés Falfán-Valencia

**Affiliations:** a HLA Laboratory, Instituto Nacional de Enfermedades Respiratorias Ismael Cosío Villegas, Mexico City, 14080, Mexico; b Departamento de Investigación en Tabaquismo y EPOC, Instituto Nacional de Enfermedades Respiratorias Ismael Cosío Villegas, México City, Mexico; c Clínica de Enfermedades Respiratorias, Fundación Médica Sur, Mexico City, 14080, Mexico

## Abstract

This article contains data on the allele and genotype frequency for single nucleotide polymorphisms (SNPs) in candidate genes *CHRNA5* (rs16969968, rs17408276, rs680244) *CHRNA3* (rs6495307, rs12914385) *NRXN1* (rs10865246, rs1882296, rs985919) and *HTR2A* (rs6311, rs6313) previously evaluated as genetic risk variants for cigarette smoking at an early age and relapse to smoking cessation treatment Pérez-Rubio et al., 2018. These SNPs were selected due to previous associations in other populations, including Mexican Mestizos. Smokers were classified according to the age at onset, cigarettes per day, nicotine dependence, COPD status and therapy received.

**Table:** Specifications table

Subject area	Genomic medicine
More specific subject area	Genetic epidemiology
Type of data	*Tables.*
How data was acquired	*Smoking pattern survey, allelic discrimination assay by real-time PCR (Applied Biosystems, Foster City, CA, USA)*
Data format	*Filtered and analyzed.*
Experimental factors	*Peripheral blood sample, DNA extraction by BDtract DNA isolation kit (Maxim Biotech, Inc. San Francisco, California, USA).*
Experimental features	*Genotyping was performed using* 3 μL *of DNA at* 15 ng/μL *concentration and TaqMan probes (Applied Biosystems Foster City CA, USA). In each template, we included 3 non-template controls, and 1% of the samples were genotyped in duplicate as an allele assignment control.*
Data source location	*HLA Laboratory, Instituto Nacional de Enfermedades Respiratorias Ismael Cosío Villegas (INER) at México City*
Data accessibility	*Accessible from this article; DNA sample and raw data are available for further analyses in collaborative studies.*

**Table:** 

**Value of the data** • Comparing and analyzing in other populations offer an opportunity to find causal genetic variants in tobacco smoking-related variables. • The analysis in mestizo smokers validates previous reports in Caucasian populations. • Further studies in other mestizo populations, with different degrees of Amerindian/Caucasian contribution, are desirables.

## 1 Data

A descriptive table (Table 1) showing characteristics on the single nucleotide polymorphisms (SNPs) is included, these comprise gene location, alleles (change/minor), chromosome position, minor allele frequency and gene consequence when it applies.

**Table 1:** Characteristics in evaluated SNPs.

Gene	SNP	Allele		Chr Position	MAF^a^	Gene Consequence^b^
		Change	Minor			
*CHRNA5*	rs16969968	G > A	A	15:78590583	A = 0.26553	Missense Variant:D [GAT] > N [AAT]
	rs17408276	T > C	C	15:78589276	C = 0.2881	Intron Variant
	rs680244	C > T	C	15:78578946	T = 0.4098	Intron Variant
*CHRNA3*	rs6495307	C > T	T	15:78597979	T = 0.3994	Intron Variant
	rs12914385	C > T	T	15:78606381	T = 0.3138	Intron Variant
*NRXN1*	rs10865246	A/C	C	2:50443116	C = 0.4950	Intron Variant
	rs1882296	T > C	C	2:50039707	C = 0.3900	Intron Variant
	rs985919	C > A	A	2:50459875	A = 0.4957	Intron Variant
*HTR2A*	rs6311	C > T	T	13:46897343	T = 0.3970	2KB Upstream Variant
	rs6313	G > A	A	13:46895805	A = 0.40701	Synonymous Variant: S [TCC] > S [TCT]

a Minor allele frequency according to The genome Aggregation Database (gnomAD).

b Codon in brackets.

Genotype frequencies for 10 SNPs in the candidate genes *CHRNA5* (rs16969968, rs17408276, rs680244), *CHRNA3* (rs6495307, rs12914385), *NRXN1* (rs10865246, rs1882296, rs985919) and *HTR2A* (rs6311, rs6313) were evaluated in the participation of smoking-related variables in Mexican Mestizo smokers and their characteristics are described in Table 2, Table 3, Table 4, Table 5.

**Table 2:** Genotype frequency of SNP in early and late smokers.

Gene	SNP/Genotype	ES (n = 81)		LS (n = 70)	
*CHRNA5*	rs16969968	n	GF (%)	n	GF (%)
	GG	41	50.6	44	63.8
	GA	34	41.9	22	31.9
	AA	6	7.4	3	4.3
	rs17408276				
	TT	48	61.5	38	55.9
	TC	29	37.2	26	37.1
	CC	1	1.3	4	5.7
	rs680244				
	GG	47	58.0	43	61.4
	GA	28	34.6	23	32.9
	AA	6	7.4	4	5.7
*CHRNA3*	rs6495307				
	CC	46	58.2	42	60.0
	CT	31	39.2	23	32.9
	TT	2	2.5	5	7.1
	rs12914385				
	CC	35	43.2	40	58.0
	CT	38	46.9	26	37.7
	TT	8	9.9	3	4.3
*NRXN1*	rs10865246				
	AA	33	40.7	27	38.6
	AC	37	45.7	38	54.3
	CC	11	13.6	5	7.1
	rs1882296				
	AA	42	51.9	39	55.7
	AG	35	43.2	26	37.1
	GG	4	4.9	5	7.1
	rs985919				
	TT	28	34.6	24	34.3
	TG	47	58.0	37	52.9
	GG	6	7.4	9	12.9
*HTR2A*	rs6311				
	CC	26	32.1	24	34.3
	CT	38	46.9	39	55.7
	TT	17	21.0	7	10.0
	rs6313				
	GG	56	69.1	60	85.7
	GA	14	17.3	5	7.1
	AA	11	13.6	5	7.1

ES: People who smoke cigarettes at an early age; LS: People who smoke cigarettes at a late age; GF: Genotype frequency.

**Table 3:** Genotype frequency of SNP in smoker status by cigarettes per day.

Gene	SNP/Genotype	HS (n = 86)		MS (n = 11)		LIS (n = 54)	
*CHRNA5*	rs16969968	n	GF (%)	n	FG (%)	n	GF (%)
	GG	47	54.7	7	63.6	31	58.5
	GA	32	37.2	4	36.4	20	37.7
	AA	7	8.1	0	0.0	2	3.8
	rs17408276						
	TT	45	54.2	5	50.0	36	67.9
	TC	36	43.4	4	40.0	15	28.3
	CC	2	2.4	1	10.0	2	3.8
	rs680244						
	GG	49	57.0	7	63.6	34	63.0
	GA	32	37.2	3	27.3	16	29.6
	AA	5	5.8	1	9.1	4	7.4
*CHRNA3*	rs6495307						
	CC	47	56.0	6	54.5	35	64.8
	CT	34	40.5	4	36.4	16	29.6
	TT	3	3.6	1	9.1	3	5.6
	rs12914385						
	CC	38	44.7	7	63.6	30	55.6
	CT	39	45.9	4	36.4	21	38.9
	TT	8	9.4	0	0.0	3	5.6
*NRXN1*	rs10865246						
	AA	33	38.4	3	27.3	24	44.4
	AC	44	51.2	7	63.6	24	44.4
	CC	9	10.5	1	9.1	6	11.1
	rs1882296						
	AA	42	48.8	6	54.5	33	61.1
	AG	37	43.0	4	36.4	20	37.0
	GG	7	8.1	1	9.1	1	1.9
	rs985919						
	TT	27	31.4	4	36.4	21	38.9
	TG	50	58.1	5	45.5	29	53.7
	GG	9	10.5	2	18.2	4	7.4
*HTR2A*	rs6311						
	CC	27	31.4	4	36.4	19	35.2
	CT	41	47.8	7	63.6	29	53.7
	TT	18	20.9	0	0.0	6	11.1
	rs6313						
	GG	69	80.2	11	100.0	36	66.7
	GA	6	8.0	0	0.0	13	24.1
	AA	11	12.8	0	0.0	5	9.2

HS: People who were heavy smokers; MS: People who were moderate smokers; LIS: People who were light smokers; GF: Genotype frequency.

**Table 4:** Genotype frequency of SNP in the level of nicotine dependence by FTCD.

Gene	SNP/Genotypes	HD (n = 63)		MD (n = 42)		LD (n = 54)	
*CHRNA5*	rs16969968	n	GF (%)	n	GF (%)	n	GF (%)
	GG	35	55.6	26	61.9	21	51.2
	GA	23	36.5	15	35.7	18	43.9
	AA	5	7.9	1	2.4	2	4.9
	rs17408276						
	TT	36	59.0	25	61.0	22	55.0
	TC	24	39.3	15	36.6	15	37.5
	CC	1	1.6	1	2.4	3	7.5
	rs680244						
	GG	33	52.4	30	71.4	23	54.8
	GA	28	44.4	10	23.8	13	31.0
	AA	2	3.2	2	4.8	6	14.3
*CHRNA3*	rs6495307						
	CC	31	50.8	28	66.7	25	59.5
	CT	28	45.9	13	31.0	13	31.0
	TT	2	3.3	1	2.4	4	9.5
	rs12914385						
	CC	32	50.8	19	45.2	21	51.2
	CT	25	39.7	20	47.6	19	46.3
	TT	6	9.5	3	7.1	1	2.4
*NRXN1*	rs10865246						
	AA	28	44.4	14	33.3	16	38.1
	AC	29	46.0	26	61.9	19	45.2
	CC	6	9.5	2	4.8	7	16.7
	rs1882296						
	AA	33	52.4	25	59.5	23	54.8
	AG	26	41.3	14	33.3	18	42.9
	GG	4	6.3	3	7.1	1	2.4
	rs985919						
	TT	25	39.7	15	35.7	11	26.2
	TG	32	50.8	23	54.8	27	64.3
	GG	6	9.5	4	9.5	4	9.5
*HTR2A*	rs6311						
	CC	19	30.2	14	33.3	17	40.5
	CT	32	50.8	24	57.1	19	45.2
	TT	12	19.0	4	9.5	6	14.3
	rs6313						
	GG	48	76.2	31	73.8	35	83.3
	GA	7	11.1	8	19.0	4	9.5
	AA	8	12.7	3	7.1	3	7.1

HD: Smokers with high nicotine dependence; MD: Smokers with moderate nicotine dependence; LD: Smokers with low nicotine dependence; GF: Genotype frequency.

**Table 5:** Genotype frequency of SNP according to COPD status.

Gene	SNP/Genotypes	COPD (n = 11)		Non-COPD (n = 140)	
*CHRNA5*	rs16969968	n	GF (%)	n	GF (%)
	GG	5	45.5	80	57.6
	GA	5	45.5	51	36.7
	AA	1	9.1	8	5.8
	rs17408276				
	TT	3	27.3	83	61.5
	TC	8	72.7	47	34.8
	CC	0	0.0	5	3.7
	rs680244				
	GG	5	45.5	85	60.7
	GA	5	45.5	46	32.9
	AA	1	9.1	9	6.4
*CHRNA3*	rs6495307				
	CC	5	45.5	83	60.1
	CT	6	54.5	48	34.8
	TT	0	0.0	7	5.1
	rs12914385				
	CC	3	30.0	72	51.4
	CT	6	60.0	58	41.4
	TT	1	10.0	10	7.1
*NRXN1*	rs10865246				
	AA	1	9.1	59	42.1
	AC	7	63.6	68	48.6
	CC	3	27.3	13	9.3
	rs1882296				
	AA	2	18.2	79	56.4
	AG	7	63.6	54	38.6
	GG	2	18.2	7	5.0
	rs985919				
	TT	3	27.3	50	35.7
	TG	6	54.5	78	55.7
	GG	2	18.2	12	8.6
*HTR2A*	rs6311				
	CC	9	81.8	43	30.7
	CT	0	0.0	75	53.6
	TT	2	18.1	22	15.7
	rs6313				
	GG	10	90.1	106	75.7
	GA	1	9.0	18	12.9
	AA	0	0.0	16	11.4

COPD: Smokers with COPD; Non-COPD: Smokers without COPD; GF: Genotype frequency.

## 2 Experimental design, materials, and methods

### 2.1 Participants

Subjects with ≥30 years, men and women. To determine Mexican Mestizo ancestry, subjects were asked about their parents and grandparents ancestry and not belong to an indigenous group (See Table 6).

**Table 6:** Genotype frequency of SNP by therapy received.

Gene	SNP/Genotypes	PT (n = 93)		CBT (n = 47)	
*CHRNA5*	rs16969968	n	GF (%)	n	GF (%)
	GG	51	55.4	28	59.6
	GA	35	38.0	17	36.2
	AA	6	6.5	2	4.3
	rs17408276				
	TT	51	58.0	29	61.7
	TC	33	37.5	17	36.2
	CC	4	4.5	1	2.1
	rs680244				
	GG	54	58.1	30	63.8
	GA	30	32.3	17	36.2
	AA	9	9.7	0	0.0
*CHRNA3*	rs6495307				
	CC	55	59.8	28	60.9
	CT	31	33.7	17	37.0
	TT	6	6.5	1	2.2
	rs12914385				
	CC	46	50.0	23	48.9
	CT	38	41.3	23	48.9
	TT	8	8.7	1	2.1
*NRXN1*	rs10865246				
	AA	35	37.6	20	42.6
	AC	47	50.5	22	46.8
	CC	11	11.8	5	10.6
	rs1882296				
	AA	47	50.5	26	55.3
	AG	43	46.2	16	34.0
	GG	3	3.2	5	10.6
	rs985919				
	TT	29	31.2	18	38.3
	TG	55	59.1	24	51.1
	GG	9	9.7	5	10.6
*HTR2A*	rs6311				
	CC	29	31.1	20	42.5
	CT	45	48.4	24	51.0
	TT	19	20.4	3	6.4
	rs6313				
	GG	68	73.1	41	87.2
	GA	13	14.0	3	6.4
	AA	12	12.2	3	6.4

PT: Smokers who received pharmacologic therapy and cognitive-behavioral therapy; CBT: Smokers who received only cognitive-behavioral therapy; GF: Genotype frequency.

Smokers recruited from the Smoking Cessation Support Clinic of the Department of Investigation in Tobacco Consumption and COPD at the Instituto Nacional de Enfermedades Respiratorias Ismael Cosío Villegas (INER) in Mexico City, with the following criteria: individuals who started smoking in adolescence (<18 years) were defined as early smokers (ES) or those who started smoking in adulthood (≥18 years) defined as late smokers (LS); these subjects have been recruited previously in our research group [2], [3].

### 2.2 Smoking-related variables

A survey with smoking-related variables was assessed to daily smokers. They were asked about their smoking pattern by cigarette smoked per day (cpd), classified as heavy smokers (HS, ≥20 cpd), moderate smokers (MS, 11–19 cpd) and light smokers (LIS, ≤ 10 cpd). Level on nicotine dependence was measured by Fagerström Test for Cigarette Dependence (FCTD), classified as high (HD), moderate (MD) and low dependence (LD) by their score as 7 to 10, 5 and 6, and less than 4, respectively. According to the intervention received by the physicians and psychologists, individuals were classified as only cognitive-behavioral therapy (CBT) without any pharmacological therapy (PT), and CBT plus any PT, which could include nicotine replacement therapy and/or Bupropion and Varenicline.

### 2.3 COPD diagnosis

As a requirement to attend the cessation sessions, participants were asked to perform spirometry in order to diagnose smoking-related diseases as chronic obstructive pulmonary disease (COPD). The diagnosis was done a physician and was based on patient medical history, physical examination, and spirometry data, with American Thoracic Society criteria. COPD was defined as an FEV1/FVC ratio of less than 70% in post-bronchodilator maneuver.

### 2.4 DNA extraction

A 15 mL peripheral blood sample in tubes with EDTA was obtained from each participant through forearm venipuncture. The DNA extraction was performed using BDtract DNA isolation kit (Maxim Biotech, Inc. San Francisco, California, USA) and later was quantified by spectrophotometry with a NanoDrop 2000 (Thermo Scientific, DE, USA). DNA samples with a concentration >100 ng/mL and purity with a 260/280 relation ≥2.

### 2.5 SNP selection

These 10 SNPs were selected because they were previously evaluated with cigarette smoking or with nicotine dependence in Mexican mestizo smokers [1].

### 2.6 Genotyping

Genotyping was performed using a real-time PCR (7300 Real-Time PCR system, Applied Biosystems, Foster City, CA, USA) based allelic discrimination assay using 3 μL of DNA at 15 ng/μL concentration and TaqMan probes (Applied Biosystems Foster City CA, USA). In each template, were included 3 non-template controls, and 1% of the samples were genotyped in duplicate as an allele assignment control. Detection Software (SDS v. 1.4, Applied Biosystems). VIC™ and FAM™ dyes were used for alleles A and B, respectively.

### 2.7 Genotype frequency

All smokers in the study were compared by full genotype frequency according to the smoking-related variables.

## Ethical approval

The protocol was approved by INER science and research bioethics and biosecurity committees (protocol number B15-16).
