# Supplementary material for: Data on genotype frequency for SNPs associated to age of smoking onset and successful smoking cessation treatment
Source: Data Brief. 2019 Apr 17;24:103893. doi: 10.1016/j.dib.2019.103893 (PMC6488734; doi:10.1016/j.dib.2019.103893)
Supplement: Supplementary file 1 — Multimedia component 1 [file mmc1.pdf]

AUTHOR DECLARATION FORM

We wish to confirm that there are no known conflicts of interest associated with this publication and there has been no significant financial support for this work that could have influenced its outcome.

We confirm that the manuscript has been read and approved by all named authors and that there are no other persons who satisfied the criteria for authorship but are not listed. We further confirm that the order of authors listed in the manuscript has been approved by all of us.

We confirm that we have given due consideration to the protection of intellectual property associated with this work and that there are no impediments to publication, including the timing of publication, with respect to intellectual property.

In so doing we confirm that we have followed the regulations of our institutions concerning intellectual property. We further confirm that any aspect of the work covered in this manuscript that has involved human patients has been conducted with the ethical approval of all relevant bodies and that such approvals are acknowledged within the manuscript.

We understand that the Corresponding Author is the sole contact for the Editorial process (including Editorial Manager and direct communications with the office). He is responsible for communicating with the other authors about progress, submissions of revisions and final approval of proofs. We confirm that we have provided a current, correct email address which is accessible by the Corresponding Author and which has been configured to accept email from (rfalfanv@iner.gob.mx).

Signed by all authors as follows:

Author	Author signature	Date of signature
Gloria Pérez-Rubio	[signature]	14/03/2019
Luis Alberto López-Flores	[signature]	14/03/2019
Alejandra Ramírez-Venegas	[signature]	14/03/2019
Raúl H Sansores	[signature]	14/03/2019.
Ramcés Falfán-Valencia	[signature]	14/03/2019.
